# Validation of a screener to assess ultra-processed food consumption in the adult Indian population: the Nova-UPF Screener (for India)

**DOI:** 10.1017/S0007114525105230

**Published:** 2025-11-14

**Authors:** Suparna Ghosh-Jerath, Srishti Mediratta, Gaurika Kumar, Sahiba Kohli, Fernanda H. Marrocos-Leite, Neha Khandpur, K. Srinath Reddy

**Affiliations:** 1 The George Institute for Global Health, New Delhi, India; 2 Lady Irwin College, University of Delhi, New Delhi, India; 3 Center for Epidemiological Research in Nutrition and Health, Faculty of Public Health, University of Sao Paulo, Sao Paulo, Brazil; 4 Wageningen University, Wageningen, the Netherlands; 5 Public Health Foundation of India, New Delhi, India

**Keywords:** Tool adaptation, Validation study, Ultra-processed foods, Nova classification, Nutrition surveys, Dietary assessment tool, Food consumption, Burden of diet-related non-communicable diseases, India

## Abstract

Increasing prevalence of diet-related non-communicable diseases in India is attributed to overconsumption of energy-dense, nutrient-poor diets and ultra processed foods (UPF) may potentially contribute to this consumption pattern. Applying standard UPF definition and developing appropriate tools can better capture its consumption among Indians. This cross-sectional study aimed to validate the ‘Nova-UPF Screener (for India)’ and explore its potential to objectively capture UPF consumption among Indian adults. The screener, adapted in prior formative research study from a tool for Brazilian population, was subjected to content, face and concurrent criterion validation. Subject matter experts (*n* 74) participated in online consultations to determine its content validity. Adults (18–60 years) from different geographical regions of India were included for face (*n* 70) and concurrent criterion (*n* 304) validations. The screener comprised twenty-four UPF categories specific to Indian food environment. Critical inputs from experts on screener’s appropriateness were incorporated to enhance its content. For face validation, overall percentage agreement of 99·4 % for all questions indicated a strong agreement for retaining screener attributes in each question. Half the participants (49·4 %) who were administered the finalised screener had Nova-UPF scores between 2 and 4 out of 24. There was almost perfect agreement (Pabak index = 0·85) between distribution of participants based on Nova-UPF scores and fifths of dietary share of UPF (as energy %) assessed by 24-h dietary recall. Nova-UPF Screener (for India) is a valid tool to capture UPF consumption in India that can be used for rapid assessment of UPF consumption and informing policies to improve Indian diets.

The prevalence of diet-related non-communicable diseases (DR-NCD) in India has been increasing over the years^(1)^. Approximately 65·9 % of deaths in India in 2019 were attributed to NCD, with diet-related factors contributing substantially to the burden of diseases such as type 2 diabetes, CVD and certain cancers^(2,3)^. Projections indicate that the overall percentage share of DR-NCD in India will increase to 58 % by the year 2051, which will further add to the health expenditures^(4)^. Consecutive rounds of National Family Health Surveys in India show rising trends of obesity and DR-NCD^(5)^. Over the years, the prevalence of overweight or obesity has risen to 24 % from 20·7 % among women and to 22·9 % from 18·9 % among men^(6,7)^. One of the biggest causes of increased DR-NCD among adults is the overconsumption of energy-dense and nutrient-poor diets^(8)^.

Nutrition transition in India is witnessing a shift in dietary patterns as people are drifting away from fresh and minimally processed foods and traditional home-cooked food to processed, packaged and ultra-processed foods that are often high in fat, sugar, salt and other ingredients such as cosmetic additives, preservatives and components that are not used in household-level culinary practices^(9–11)^. The limited data on dietary intake at the population level in India^(12)^ show a deficiency of essential food groups such as pulses, milk and milk products, fruits and vegetables, whereas consumption data of packaged foods such as chips, biscuits, chocolates, sweets and sugar-sweetened beverages show an increasing trend^(13)^. The Euromonitor (2020) data show that the overall per capita sales of packaged and processed foods in India nearly doubled from USD 31·3 in 2012 to USD 57·7 in 2018, with its consumption not being restricted to metropolitan cities but also spreading to smaller cities of the country^(14,15)^.

Different terminology has been applied to capture this range of packaged and processed foods, including junk foods, foods that are high in fat, sugar and salt, processed foods and ultra-processed foods (UPF). According to the Nova food classification system that categorises foods based on the purpose and the level of processing, UPF are a category of foods that undergo a series of industrial processes such as extrusion and moulding and have the presence of classes of additives such as flavours, flavour enhancers, colours, emulsifiers, thickeners and sweeteners whose function is to make the final product hyperpalatable or more appealing, Although not unique to UPF, they often include additives that prolong the product duration (shelf life) and protect original properties or prevent the proliferation of microorganisms^(16–19)^. Ready-to-consume packaged products like carbonated soft drinks and extruded snacks such as chips, chocolates, confectionery, ice creams and desserts, bread, spreads, biscuits, cakes, breakfast cereals, fruit drinks, pre-prepared ready-to-cook foods, instant soups and noodles are some common UPF^(19)^. These foods often present nutrient-poor profiles (e.g. high in fat, sugar and salt and low in dietary fibre, micronutrients and vitamins) and may have potential contaminants from packaging material and processing^(18)^. Dietary patterns rich in UPF have been associated with increased rates of obesity, development of DR-NCD such as dyslipidaemias, high blood pressure, hyperglycemia and premature mortality globally^(20–23)^. India is experiencing a ‘double burden of malnutrition’^(24,25)^ indicating the coexistence of undernutrition and overnutrition (overweight and obesity) with a rise in DR-NCD. In such a scenario, it becomes imperative to first develop a tool to systematically assess UPF consumption and then understand its role in increasing the burden of DR-NCD and all forms of malnutrition.

Despite a growing body of evidence supporting the amplified dietary share of UPF as a potent indicator of poor diet quality,^(26,27)^ there is a lack of comparable data on UPF consumption across contexts and over time, especially in low- and middle-income countries. National-level data are not readily available because assessing the dietary contribution of UPF using quantitative 24-h dietary recall or semi-quantitative food frequency data is expensive and time consuming^(28)^. Thus, the development of a simple and quick dietary screener with a low respondent and researcher burden for estimating UPF consumption across countries is essential^(29)^. One such dietary screener has been developed in Brazil^(29)^ called the ‘Nova-UPF Screener (for Brazil)’, which uses the Nova food classification^(19)^.

The present study aimed to adapt and validate a tool called the ‘Nova-UPF Screener (for India)’. The key output of the study was a validated screener in Hindi and English that would use a scoring system to quantify the individual-level UPF consumption among Indian adult consumers.

## Methods

### Study design

The screener developed during the formative phase^(30)^ was presented to domain experts during six online consultations using purposive sampling for content validation. Following this, the revised screener was finalised in English language and translated into Hindi language for face and concurrent criterion validation using a cross-sectional survey.

### Development of the Nova-UPF Screener (for India)

**Figure 1.**
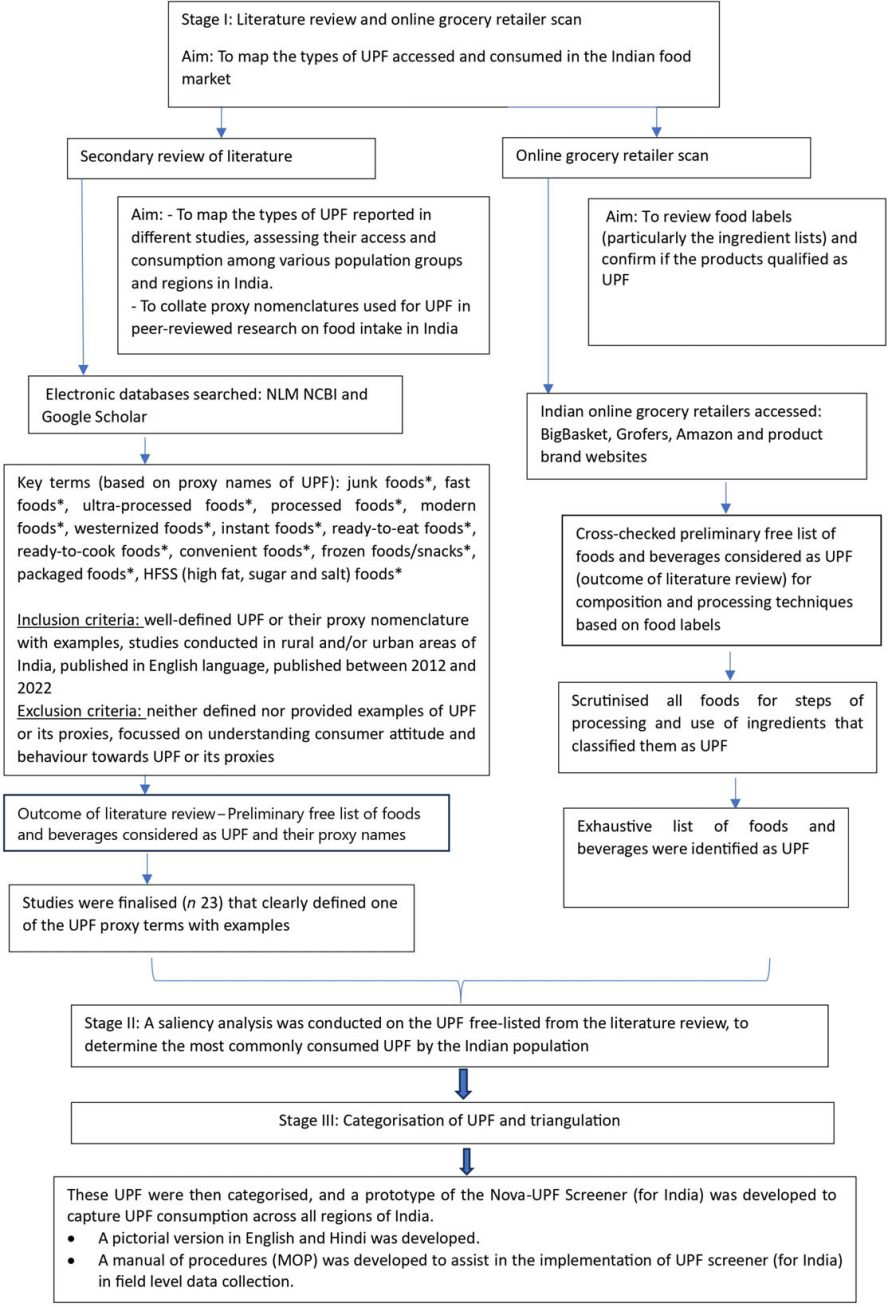
Adaptation of the Nova-UPF Screener (for India).

**Figure 2.**
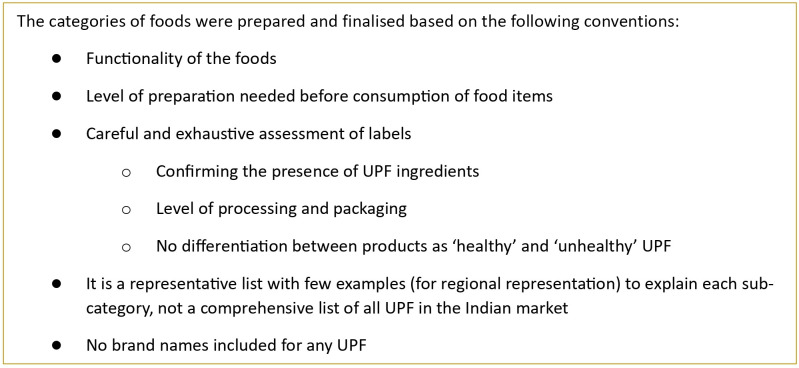
Conventions followed for classifying UPF into categories for the Nova-UPF Screener (for India).

To develop the Nova-UPF Screener (for India), we conducted a formative research study involving three steps that identified a list of UPF relevant to the Indian context (Figure 1). Step 1 included an extensive review of the published literature on UPF accessed and consumed in India. The list of UPF generated was supplemented by an online grocery retailer scan in Step 2 of the study, where the food ingredients listed on the packaging labels were checked to see if they qualified as UPF ingredients. Foods containing additives (such as flavour enhancers, colours and emulsifiers) and industrially derived modified sugars, proteins and fats were confirmed to be ultra-processed. In Step 3, UPF thus identified were free-listed, and a saliency analysis was performed to understand the preference ranking of the UPF categories among the Indian population. The detailed study^(30)^ is available that comprehensively explains the three steps involved in mapping of UPF in the Indian context. After generating a list of UPF most commonly accessed and consumed in India, a list of conventions (such as checking the functionality of UPF, i.e. it is a snack, an ingredient and the level of preparation needed before consumption) was set to categorise the UPF and develop the first draft of the Nova-UPF Screener (for India) in English (Figure 2). The Nova-UPF Screener, thus developed, was then validated through content^(31)^, face^(32)^ and concurrent criterion validation^(33)^ techniques.

### Validation of the Nova-UPF Screener (for India)

#### Content validation

The content validation was conducted to obtain inputs from subject matter experts on the content of the screener. Online virtual consultations at the regional and national levels were organised for this purpose.

##### Study participants and study locale

Regional subject matter experts, comprising academics, public health professionals, dieticians, food technologists and food and nutrition scientists, were identified and invited through electronic mail invitations to take part in six consultations conducted across five regions of India. A total of sixty-four experts were invited, out of which fifty-four experts accepted the invitation and attended the respective regional consultations. The experts belonged to the states/ union territories of Delhi, Haryana, Punjab and Jammu and Kashmir from the North region (*n* 9); West Bengal from the East region (*n* 6); Assam, Meghalaya and Manipur from North-East region (*n* 14); Gujarat, Maharashtra, Madhya Pradesh, Rajasthan from West region (*n* 17) and Tamil Nadu, Kerala, Telangana, Karnataka from South region (*n* 8). National-level experts (*n* 20 invited, 13 attended) included key stakeholders from government bodies, non-governmental organisations, academia, public health experts, food technologists and international collaborators of this study.

##### Data collection

Regional group consultations were carried out virtually between February and April 2022, wherein a detailed presentation on the study’s background, rationale, objectives and purpose of the consultations was made. The screener in English was presented, followed by detailed deliberations on a set of questions about the screener’s content, length, language, regional representation and ease and modality of implementation. The experts provided their views through the questions (online Supplementary Table 1), which helped the study team make further revisions to the screener. Subsequently, a national consultation was convened virtually in May 2022 to present and deliberate on compiled suggestions from regional consultations. The practical considerations for including the tool as part of a national nutrition survey were also discussed at the national consultation. The expert views are presented in the supplementary table (online Supplementary Table 2). The screener was further modified, translated into Hindi and converted into a pictorial tool. Pictures, illustrations and/or food emoticons of some of the examples from each UPF category were used for a better understanding of the UPF categories. It was then subjected to face and concurrent criterion validation.

#### Face validation

In face validation, the screener was assessed for its appropriateness in capturing the consumption of UPF in the Indian population. The structure, appearance and design of the screener were assessed along with detailed inputs on clarity and ease of understanding. This was done using subjective and objective face validation techniques (as detailed below).

##### Study participants and study locale

For subjective face validation, the screener was administered to adults (*n* 50) aged 18–60 years, purposively selected from diverse geographic and demographic (urban, rural and peri-urban) strata. Consenting participants belonged to five states of India, namely, Maharashtra, Jharkhand, Uttarakhand, Madhya Pradesh and Delhi National Capital Region. The participants included nearly equal representation of males and females. For objective face validation, the screener was administered to purposively selected adults (*n* 20) aged 18–60 years with an equal representation of males and females, ten each from Delhi National Capital Region (Gurugram) and Andaman and Nicobar Islands, a union territory.

##### Data collection

Screener translated after content validation was back translated to ensure identical capture of information in both English and Hindi. After taking consent, the screener was administered to each participant based on their language preference (English or Hindi), to obtain their inputs on various aspects of the screener through a one-on-one interview. Participants were asked to provide detailed inputs on the screener’s structure, appearance and design and on the clarity, ease of understanding and interpretation of the list of UPF categories. For objective face validation, the screener was adapted to a digital platform using the Census and Survey Processing System software (version 7.7). Based on the language preference (English or Hindi) of the participants, an interviewer administered the screener to assess the participants’ understanding of instructions, the appropriateness of sub-categories, pictures and examples in the screener using a questionnaire (online Supplementary Table 3).

#### Concurrent criterion validation

The concurrent criterion validation was conducted to test the agreement of the scores obtained using the screener against the calorie share of UPF consumed using a 24-h dietary recall, a gold standard method for dietary intake assessment. Consenting adults (18–60 years) were conveniently selected from diverse geographic, socio-economic and demographic strata from three states, that is, Delhi-National Capital Region, Jharkhand and Madhya Pradesh.

##### Sample size calculation

The sample size for concurrent criterion validation was calculated based on the proportion of adults in urban India (73 %) purchasing processed foods and beverages^(34)^. The sample size was calculated to be 304, with a precision of 5 % and a 95 % level of significance using the following formula.

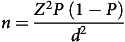




##### Data collection

The study participants were informed about the purpose of the study through a participant information sheet that was explained by the field team and invited to participate in the study. Those who agreed to participate were asked to sign an informed consent sheet. The screener was then administered to them based on their language preference, English or Hindi, followed by assessment of their dietary intake using the 24-h dietary recall method. This 24-h recall survey form was pre-tested and administered by the nutritionist. The screener was administered before the 24-h dietary recall to avoid potential bias that could result from the prior application of a more detailed tool. During the administration of the screener, all twenty-four UPF categories were read out to the participants. Participants responded affirmatively as ‘yes’ if they consumed any food/drink item(s) from the example list of items comprising the UPF categories, in the past 24 h, which was explained to the participants as ‘the time period between the time they woke up in the morning yesterday to the time they went to bed at night to sleep yesterday’. For assessing their full day’s dietary intake using the 24-h dietary recall, participants were asked to recall and report details of their complete food intake for each meal consumed during the past 24 h. In order to facilitate this recall and portion size estimation, a culturally appropriate flip book with pictorial representations of portion sizes of various food items (fruits, vegetables, local fresh foods and locally available processed and ultra-processed foods) was developed and presented to participants. This addressed possible recall bias and provided a standardised estimate of dietary intake. Standard measuring cups and spoons were also used for estimating intakes in household measures. A detailed description of home-cooked mixed dish recipes with weight estimates of each ingredient (brand name, in case of UPF), total amount prepared and portion of the total recipe consumed was elicited and recorded. Details of the food eaten outside the home were also prompted and recorded.

### Data analysis

Experts’ suggestions from regional and national consultations for assessing the screener contents were collated and thoroughly reviewed for UPF characteristics. The suggestions were listed, and each suggestion was handled as ‘to be incorporated’ and ‘no change was needed’, based on the votes received for each suggestion or based on the interpretation of the transcript of the discussion. Accordingly, all necessary changes were incorporated into the screener. This finalised version of the screener was presented for subjective face validation, where inputs from study participants were qualitatively analysed. For objective face validation, the percentage agreement for each question and overall agreement were quantitatively measured using the formulas given below. The screener’s attributes asked through these questions were retained when a percentage agreement of more than 90 % was reached, indicating full strength of agreement.

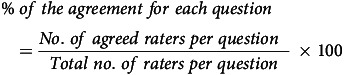




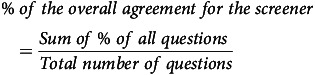




For concurrent criterion validation, firstly, the Nova-UPF score for each participant was calculated by the total number of UPF categories consumed. Each consumed category was scored one point, and the scores could range from 0 to 24. The 24-h dietary recall sheets were cleaned for entry errors and inconsistencies. Cleaned data were then integrated into DietCal Pro Survey Software (Version 12.0, Profound Tech Solution) for further analysis to estimate the dietary share of UPF in the day’s diet. Each food item reported in the 24-h dietary recall was initially classified into UPF or non-UPF categories based on the same criteria used for characterising the UPF as used for developing the UPF screener. Then, the consumed quantity of each item, reported in household measures, was transformed into grams and converted into calories using data from Indian Food Composition Tables^(35)^ along with the data on nutritive values of processed and ultra-processed foods available on the food labels or from secondary literature. The percentage of calories obtained from UPF consumption reported in the 24-h recall was calculated and compared with the Nova-UPF scores. The variation in the average percentage of calories from UPF according to the score variation was expressed continuously and at intervals corresponding to fifths (quintiles) of their distributions. In both cases, linear regression models were used to test the linear trends. The degree of agreement between the fifths (quintiles) of Nova-UPF scores and the fifth (quintiles) of the percentage of calories shared from UPF was evaluated by calculating the prevalence-adjusted and bias-adjusted kappa (Pabak) index^(36)^. For the Pabak index, values greater than 0·80 indicate an almost perfect agreement; between 0·61 and 0·80, a substantial agreement; between 0·41 and 0·60, moderate; between 0·21 and 0·40, fair; and equal to or less than 0·20, slight agreement^(29,37)^. The variation in the prevalence of relatively high consumption of UPF according to age group was assessed using two criteria (i) consumption equivalent observed in the upper fifth of the distribution of UPF screener scores (≥ 5) and (ii) consumption equivalent observed in the upper fifth of the distribution of the percentage share of calories from UPF (≥ 25·02 %). Pearson’s correlation was used to assess associations. Data analyses were performed with the Stata® 16.1 software, and the Pabak index was calculated using R Studio software.

## Results

### Development of the Nova-UPF Screener (for India)

The UPF screener was adapted from the Brazilian version using a review of the literature and an online Indian grocery retailer scan. A list of UPF commonly accessed and consumed by the Indian population was developed. This was followed by a saliency analysis for ranking the UPF based on their preferences among the Indian population as reported in the literature reviewed (Figure 1). The first draft of the Nova-UPF Screener (for India) was developed, which included twenty-four UPF categories along with a pictorial version of the screener to bring more clarity to the UPF included in the screener categories. A manual of procedures was developed to support training for administering the screener in the field during the validation process. The manual of procedures also included an exhaustive list of UPF examples for each category. After adaptation^(30)^, the Nova-UPF Screener (for India) was validated for its suitability in the Indian context.

### Validation of the Nova-UPF Screener (for India)

#### Content validation

Experts who participated in the regional consultations suggested the inclusion of various packaged traditional regional foods that have UPF equivalents available (online Supplementary Table 4). Only those packaged traditional food products were included in the screener that complied with the UPF definition of the Nova food classification system^(16–19)^.

**Figure 3.**
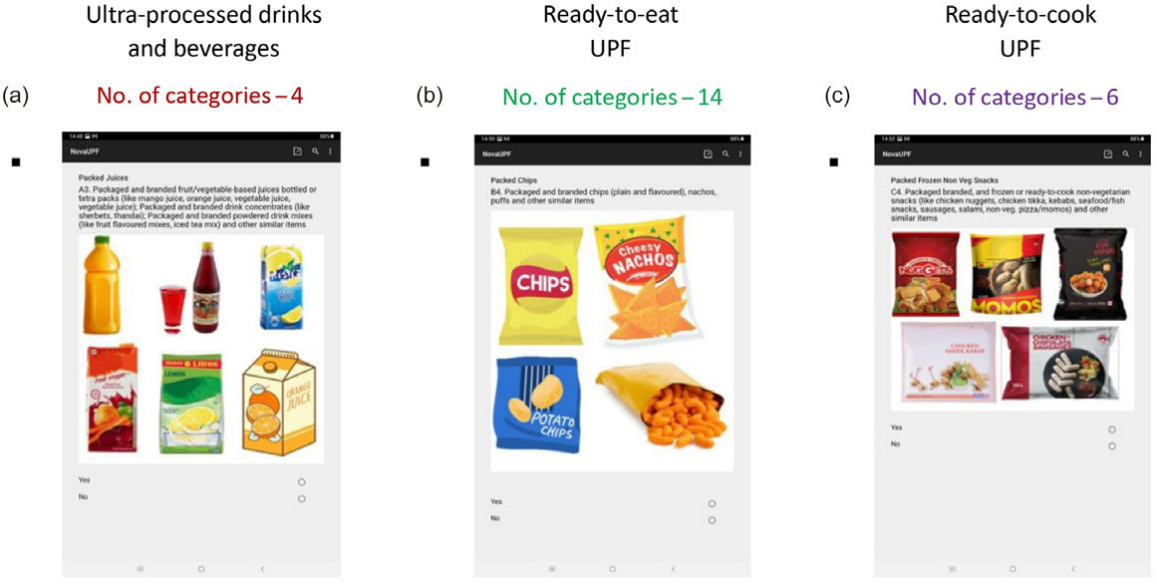
Final sections included in the Nova-UPF Screener (for India).

Certain suggestions like alcoholic beverages and ‘*paan masalas*’ (a traditional mouth freshener which sometimes contains chewable tobacco) were not included in the list because of their negative health effects independent of that of the dietary pattern and were deemed to be beyond the scope of the screener. Experts recommended the addition of food items such as pizzas, burgers, French fries, momos and other commonly consumed ready-to-eat commercial fast-foods under a specific section of the screener. They also appreciated the use of pictorial depiction for better comprehension of each UPF category and reiterated the refrain from displaying any brand names. The contents of manual of procedures for the screener were enriched by the experts’ suggestions. The length of the screener was considered appropriate by the experts. Changes were recommended for renaming certain UPF categories (Table 1). Further, for ease of understanding, the screener was divided into three major sections (Figure 3):

**Table 1.** Input from regional consultation experts on the draft categories of the Nova-UPF Screener (for India) during content validation

Recommended changes in the UPF categories	Revised categories based on the recommendations
The energy drinks sub-category (such as Redbull and Gatorade) should be renamed to avoid confusion with malted health drink powders, such as Bournvita, as these malted health drinks are also marketed as energy drinks	The energy drinks sub-category was revised to ‘Energy/Sports drinks (like clear drinks with added electrolytes, vitamins, minerals)’ for better clarity.
Commercial malt-based beverages should not be labelled as milk-based powdered health drinks.	Milk-based powdered health drinks were revised to milk or malt-based powdered health drinks.
The term ‘extruded’ should be added to the breakfast cereal subcategory.	The breakfast cereal subcategory was revised to packaged, and branded extruded/coated breakfast cereals.

1. Section A – Ultra-processed drinks and beverages

2. Section B – Ready-to-eat UPF, no or minimal preparation needed

3. Section C – Ready-to-cook UPF

During the national consultation, experts shared their inputs on the content of the revised screener and its mode of administration. More UPF, such as protein powders and peanut butter, were added to the screener after verification, owing to their increased consumption among Indians. Experts agreed upon the feasibility of incorporating the UPF screener as an additional module in national nutrition surveys. The need for its translation into regional languages for application across India was underscored. Owing to the differential literacy rate in India, for administering the screener as part of the national survey, a face-to-face interview using an Android-based application was suggested as the preferred mode of administration of the screener.

#### Face validation

In face validation, views were invited regarding the ease of understanding and interpretation of the categories of UPF in the screener. Terms such as ‘drink concentrates’, ‘powdered mixes’, ‘margarine’ and ‘instant noodles’ were poorly understood. Participants from the rural and peri-urban areas could not understand the term ‘yoghurt’ mentioned in one of the categories. For better understanding, specific terms were revised, or appropriate examples were added to the screener and the manual of procedures for the screener (Table 2). Objective face validation resulted in a 100 % agreement on seven out of the eight questions regarding clarity of instructions, sub-categories, pictures, regional representation and length of the screener. One of the questions, ‘Were the examples given in each sub-category enough?’ achieved a percentage agreement of 95 %. All the questions had a percentage agreement exceeding 90 % demonstrating the full strength of the agreement, and each attribute pertaining to the questions was retained as such. The overall percentage agreement for the screener was 99·4 % (a strong agreement).

**Table 2.** Participant observations during face validation and counter changes made in the Nova-UPF Screener (for India)

Observations	Counter changes made in the screener
The ‘flavoured water’ term was neither understood by many participants nor was it consumed.	‘Flavoured water’ removed from sub-category – aerated/ cold/ soft drinks.
The terms ‘drink concentrates’, ‘powdered mixes’, ‘margarine’ and ‘instant noodles’ were not understood by the participants.	Brand names were used for easy recall of the products. These names were added in the MOP but were not presented as part of the screener.
The participants from rural and peri-urban areas were not able to relate to the term ‘yoghurt’.	Along with ‘yoghurt’, terms such as ‘kheer’ and ‘payasam’ are also used as indicative examples of the food category.
‘Jelly toffees’ were perceived to be a part of the ‘jams, marmalades and jellies’ sub-category instead of the ‘chocolates’ sub-category.	‘Aam papad/fruit leather/bars’ was removed from the ‘jams, marmalades and jellies’ sub-category and added to the ‘chocolates’ sub-category.

MOP, manual of procedures.

#### Concurrent criterion validation

The final version of the Nova-UPF Screener (for India), comprising twenty-four UPF categories, was subjected to concurrent criterion validation. The screener was administered to 304 participants, followed by the administration of a 24-h dietary recall questionnaire. The mean age of the participants was 30·1 (sd 10·4) years; the majority of whom had completed schooling (92 %), (i.e. those who completed schooling till the highest class of a secondary school). These participants belonged to urban areas (97 %) of Delhi-NCR, Ranchi, in the state of Jharkhand and Jabalpur in the state of Madhya Pradesh. Most participants (58 %) were salaried employees, and one-third (35 %) were college students (Table 3). Table 4 shows the consumption frequency of the UPF based on the participants’ responses to the screener, depicting that most participants (44 %) consumed ‘packaged and branded biscuits, cream biscuits, cookies and cream puffs/rolls. Additionally, approximately one-third of the participants (36 %) reported consuming ‘packaged and branded ketchup, chutneys/instant chutney powders/tastemakers; packaged and branded pickles, sauces, instant gravies/curries/pastes’ and 30 % consumed ‘packaged and branded bread’. One-fourth of the participants (26·3 %) consumed ‘packaged and branded Indian *namkeens* (traditional Indian Savoury snacks)’ and 23·4 % reported intakes of ‘packaged and branded flavoured milk; packaged and branded milk or malt-based powdered health drinks, protein powder; packaged and branded yoghurt/curd-based drinks; packaged and branded milk substitutes and ready-to-drink tea/coffee mixes and dairy whiteners’ (Table 4).

**Table 3.** Socio-demographic characteristics of study participants of the concurrent criterion validation of the Nova-UPF Screener (for India) (*n* 304)

Socio-demographic variables	*n*	%
Gender		
Male	172	56·6
Female	132	43·4
Age (years)		
18–24	118	38·8
25–34	98	32·2
35–44	48	15·8
45–54	33	10·9
55–59	7	2·3
Mean, years	30·1	
sd, years	10·4	
Study site		
Delhi-NCR	107	35·2
Ranchi	102	33·6
Jabalpur	95	31·3
Locality		
Urban	294	96·7
Rural	10	3·3
Educational qualification		
Primary and lower	8	2·6
Lower than secondary	16	5·3
Secondary and completed schooling	103	33·9
Diploma	5	1·6
Graduate and above	172	56·6
Occupation		
Unemployed	3	1·0
Homemaker	3	1·0
Student	107	35·2
Salaried	176	57·9
Business	14	4·6
Self employed	1	0·3
Family type		
Staying alone	68	22·4
Nuclear	159	52·3
Joint	77	25·3
Monthly family income (INR)		
≤ 8333	28	9·2
8333–16 667	56	18·4
16 667–41 667	75	24·7
41 667–83 333	57	18·8
≥ 83 333	88	28·9

NCR, National Capital Region.

Monthly household income classes were constructed based on data from the Consumer Pyramids Household Survey (CPHS), conducted by the Centre for Monitoring Indian Economy (CMIE). Income groups were categorised into fixed brackets for ease of interpretation.

**Table 4.** Consumption frequency (%) of ultra-processed foods (UPF) on the day prior to the interview among participants (*n* 304) based on 24 UPF categories of the screener

Ultra-processed foods	UPF Section	Consumption frequency	
		*n*	%
Packaged and branded biscuits, cream biscuits, cookies and cream puffs/rolls	Section B	133	43·8
Packaged and branded ketchup, chutneys/instant chutney powders/tastemakers (such as tamarind chutney, green chutney and lemon rice spice mix); packaged and branded pickles (veg. /non-veg.), sauces (like pasta-pizza sauce), instant gravies/curries/pastes (such as mustard paste, puliyogare paste and ginger-garlic paste)	Section B	108	35·5
Packaged and branded bread (such as sliced bread, pao, burger buns, pizza base and tortillas)	Section B	91	29·9
Chocolates; toffees; lollipops; chewing gums; fruit candies; *Aam papad*/ Fruit leathers/ bars; flavoured mouth fresheners	Section B	88	28·9
Packaged and branded Indian *namkeens* (such as *aloo bhujia,* mixtures, *murukku,* flavoured and coated nuts)	Section B	80	26·3
Packaged and branded flavoured milk (like chocolate milk); packaged and branded milk or malt-based powdered health drinks, protein powder; packaged and branded yoghurt/curd-based drinks (like flavoured *lassi,* probiotic drinks); packaged and branded milk substitutes (such as soymilk and almond milk); ready-to-drink tea/coffee mixes (such as masala tea and choco mocha); dairy whiteners	Section A	71	23·4
Packaged and branded chips (plain and flavoured), nachos and puffs	Section B	47	15·5
Packaged and branded milk-based spreads, mayonnaise, dips, cheese products (such as cheese slices/cubes/spreads), salad dressings, nut spreads (such as hazelnut spread and peanut butter), chocolate spreads	Section B	36	11·8
Packaged and branded, bottled or tetra packs fruit/vegetable-based juices (like mango juice, orange juice and vegetable juice); packaged and branded drink concentrates (such as *sherbets* and *thandai);* packaged and branded powdered drink mixes (such as fruit flavoured mixes and iced tea mix)	Section A	34	11·2
Aerated/cold/soft drinks; diet drinks	Section A	31	10·2
Packaged and branded ice creams, flavoured ice bars and *kulfi*	Section B	30	9·9
Packaged and branded cakes, muffins, waffles and donuts; packaged and branded Indian sweets (such as flavoured *sonpapadi* and *gulab jamun)*	Section B	28	9·2
Packaged and branded instant soups, instant noodles/ pasta	Section C	23	9·2
Packaged and branded fruit-based preserves (such as jams, marmalades and jellies)	Section B	22	7·2
Packaged and branded extruded coated breakfast cereals (such as sugar-coated cornflakes, chocolate breakfast cereals and ragi bites); packaged and branded cereal bars (such as granola bars and energy bars)	Section B	11	3·6
Packaged, branded and flavoured yoghurt, fruit yoghurt and *kheer/payasam*	Section B	5	1·6
Packaged and branded bread mixes; packaged and branded dessert mixes (like jelly, custard, ice cream, *gulab jamun,* cake, brownie and pancake)	Section C	5	1·6
Pizza, burgers, French fries and wraps from fast-food chains	Section B	4	1·3
Energy drinks; sports drinks (like clear drinks with added electrolytes, vitamins and minerals)	Section A	3	1·0
Packaged and branded instant dishes/snacks (such as *poha, upma* and savoury oats); Packaged and branded ready-to-cook powdered mixes (such as *idli, dosa, dhokla, aloo bonda* and *dahi vada)*	Section C	3	1·0
Packaged, branded and frozen ready-to-cook non-vegetarian snacks (like chicken nuggets, chicken tikka, kebabs, seafood/fish snacks, sausages, salami and non-veg. pizza/momos)	Section C	3	1·0
Margarine; Packaged and branded flavoured butter (like garlic butter), coconut cream/milk	Section B	2	0·7
Packaged, branded and frozen ready-to-cook vegetarian snacks (like French fries, paneer snacks, vegetarian burger patty, *aloo tikki* and veg. *samosas/*pizza); Packaged and branded frozen *parathas,* puff pastry/spring roll sheets	Section C	2	0·7
Packaged and branded ready-to-cook meals in cups (such as *rajma chawal, biryani, idli sambar, bisi-belle bath* and schezwan rice)	Section C	0	0·0

Section A, UPF drinks and beverages; Section B, ready-to-eat UPF; Section C, ready-to-cook UPF.

**Figure 4.**
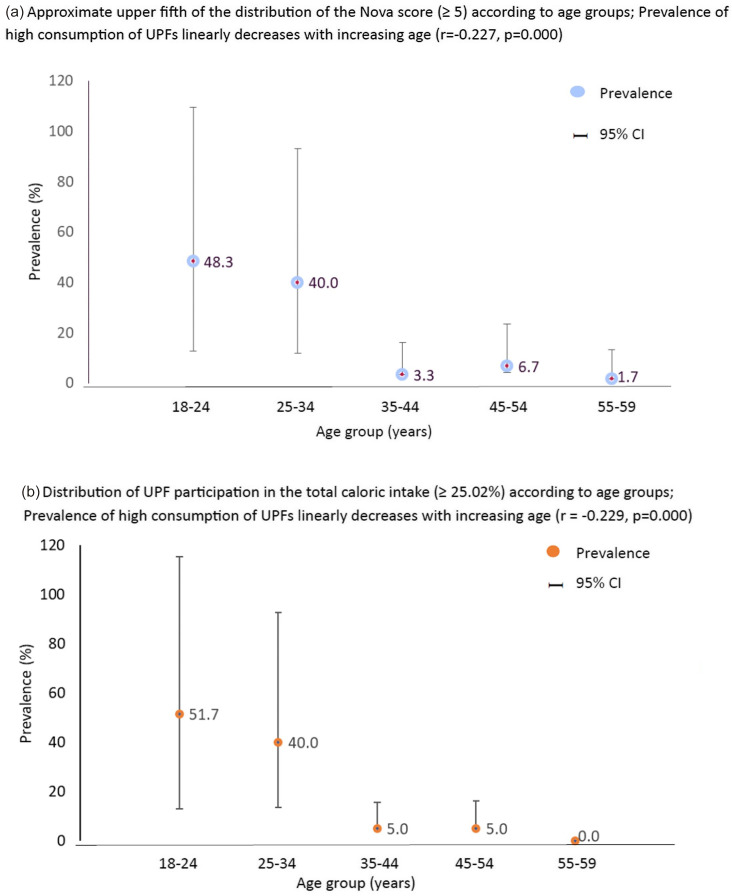
Variation in the prevalence (%) of high consumption of ultra-processed foods according to age group based on Nova Score (≥ 5) and total caloric intake (≥ 25·02 %) among participants (*n* 304).

The average Nova-UPF scores among participants were 3 (sd 2·2) (range 0–12). Most participants obtained a score of 2 (19·1 %), followed by 1 (18·1 %), 3 (17·1 %) and 4 (13·2 %), while 13 % of the participants did not consume any UPF on the previous day. Table 5 shows the distribution of Nova-UPF scores among participants and the percentage energy share from UPF in the diet. On measuring the linear correlation between the Nova-UPF score and dietary share of UPF, a strong positive correlation was observed (*r* = 0·76, *P* < 0·001). Most participants (19·1 %) had a dietary share of UPF contributing to 11·1 % of the total day’s calorie intake. The distribution of participants (Table 6) based on their classification according to the fifths of the dietary share of UPF and Nova-UPF scores showed ‘an almost perfect agreement’ (Pabak index = 0·85). Figure 4 presents the variation in prevalence of high UPF consumption according to age group based on Nova Score (≥ 5) and total caloric intake (≥ 25·02 %) among participants. The prevalence of relatively high consumption of UPF in the approximate upper fifth distribution of the Nova-UPF scores (≥ 5) and upper fifth distribution of UPF per cent share in the total caloric intake (≥ 25·02 %) linearly decreased with increasing age (*r* = –0·227, *P* < 0·001 and *r* = –0·229, *P* < 0·001, respectively).

**Table 5.** Nova scores, % population and corresponding dietary share of ultra-processed foods based on 24-h dietary recall (*n* 304)

Scoring for consumption of ultra-processed foods (obtained from the screener)	Sample		Dietary share of ultra-processed foods (% of total energy)	
	*n*	%	Average (obtained from 24-h recall data)	95 % CI
0	39	12·8	0·0	0·0, 0·0
1	55	18·1	6·7	5·3, 8·6
2	58	19·1	11·1	9·2, 12·9
3	52	17·1	14·2	11·9, 16·5
4	40	13·2	22·2	19·8, 24·9
5	27	8·9	22	19·1, 24·3
6	13	4·3	25·7	19·2, 32
7	11	3·6	32·1	28·5, 35·2
8	3	1·0	24·5	14·5, 33·4
9	3	1·0	42	32·1, 56·7
10	2	0·7	37·9	32·7, 43·2
11	0	0·0	0·0	0·0, 0·0
12	1	0·3	30·6	30·6, 30·6

**Table 6.** Distribution of participants (%) by UPF energy contribution quintiles (from 24-h dietary recall) and Nova-UPF score quintile (*n* 304)

Quintiles of dietary share of UPF from 24-hour recall (% of total calories)	Nova score quintiles for the consumption of UPF					
	Q1(0–1)	Q2 (2)	Q3 (3)	Q4 (4)	Q5 (5 or +)	Freq (%) Total
Q1 (≤ 6·2)	**22·7**	4·6	3·0	0·7	0·3	31·3
Q2 (6·3–11·3)	5·6	**6·9**	4·3	1·3	0·7	18·7
Q3 (11·4–17·7)	2·3	4·6	**4·9**	2·3	3·0	17·1
Q4 (17·8–25·0)	0·0	1·6	3·6	**3·0**	4·9	13·2
Q5 (≥ 25·1)	0·3	1·3	1·3	5·9	**10·9**	19·7
Total	30·9	19·1	17·1	13·2	19·7	**100**

UPF, ultra processed foods.

Pabak index (prevalence-adjusted bias-adjusted Kappa) = 0·85 (0·75–0·95).

Quintiles were made by dividing the variable into five equal parts using percentile points (P_20_, P_40_, P_60_ and P_80_) as the cut-off values.

## Discussion

This paper presents the findings of a validation study for a screener that captures the consumption of UPF in the Indian population. The Nova-UPF Screener (for India) was developed to suit the Indian context and then validated. The content validation provided critical inputs from regional and national level experts on the screener’s content, length, language, regional representation and ease and modality of implementation. Face validation provided insights into participants’ acceptance and understanding of the tool and also assured a strong agreement of 99·4 % for the screener. The concurrent criterion validation showed an almost perfect agreement between the distribution of participants based on the fifths of the dietary share of UPF and fifths of Nova-UPF scores (Pabak index = 0·85). The major consumption of UPF among the study population was reported from packaged and branded biscuits, breads, sauces and ketchups, chocolates, toffees and Indian savouries (namkeens).

Only a few studies^(38–40)^, to our knowledge, have reported the consumption of UPF in India, which could be attributed to the lack of uniform application of the classification of UPF as a food category in the literature on the Indian food environment. This study fills a critical gap by developing a screener that captures UPF consumption in the Indian context and validating it. The data obtained using the screener can provide some critical information to address policies trying to tackle the risk factors of the rising burden of DR-NCD in India, with the transitioning food environment being one of them. At present, most of the literature and policy documents have used ‘high in fat, sugar, and salt foods’ even though many such foods are ultra-processed in nature^(41,42)^. Recently, in May 2024, the National Institute of Nutrition – Indian Council of Medical Research (NIN-ICMR) released the UPF definition in Dietary Guidelines for Indians, 2024^(43)^.

Since efforts were made not only to develop a new tool (a quick screener) to assess UPF consumption in India but also to coherently apply the term UPF to assess its consumption in India, it was critical to validate this tool meticulously and systematically for its content. This was an important step to ensure the development of a high-quality novel tool that could be used in nutrition research^(31,44)^ and also inform policies around regulating the food environment to prevent DR-NCD. The content validation exercise of the study provided a robust review of the tool by capturing the perceptions and views of experts working in this domain. The process not only evaluated the contents of the screener but also its applicability in a diverse country like India. Additionally, the screener’s comprehensibility is critical for its utilisation as a research tool to assess UPF consumption and a study instrument for any sentinel survey across India. Thus, ensuring face validation is an important step in establishing the overall validation of this assessment tool^(32)^.

Validation by comparing the Nova-UPF scores with the dietary share of UPF using one of the gold standard methods of dietary assessment revealed an almost perfect agreement (Pabak index = 0·85). Our findings were similar to the earlier study, where the parent UPF screener developed for the Brazilian population showed significant agreement (Pabak index = 0·67) between the screener scores and the dietary share of UPF assessed using 24-h dietary recall^(29)^. This original screener, after due adaptation, was also validated in Senegal, where a near-perfect agreement was observed between the UPF score and the UPF dietary share, with a Pabak index of 0·84^(45)^.

In the present study, a fifth of the participants (19·1 %) had an average dietary share of UPF contributing to 11·1 % of the total energy intake in a day. The National Nutrition Monitoring Bureau survey conducted in India also highlighted that the intake of unhealthy packaged foods, mostly ultra-processed foods, such as chips, biscuits, chocolates, sweets and juices, among urban adults contributed to 11 % of their total energy intake^(13)^ which is lower in comparison to developed nations. Studies conducted in developed nations have shown that the intake of UPF contributed to 25 %, 42 % and 58 % of total energy intake in the diets of adults in Korea, Australia and the USA, respectively^(27,46,47)^. Akin to our results, evidence has shown that the consumption of UPF linearly decreases with increasing age^(48)^. Studies have also reported that the factors affecting the consumption of UPF, such as the lack of time to prepare foods and the lack of motivation/willpower to eat healthy, reduce as age increases^(49–51)^. Nutrition transition in low- and middle-income countries such as India is witnessing a rise in the consumption of packaged ultra-processed foods that are high in fat, sugar and/or salt^(20,34,52)^. Overconsumption of such UPF is associated with obesity and risk of developing many DR-NCD, including diabetes mellitus, CHD and cancers in adults^(53–56)^. Timely and appropriate interventions can slow down the rise in UPF consumption in low- and middle-income countries such as India^(57)^. Even though studies have shown that overconsumption of ultra-processed food leads to DR-NCD, comparable data are scarce on UPF intake in the Indian context. The screener developed and validated in the present study, however, needs to be validated in other population groups, such as children < 5 and adolescents.

The data obtained from screeners developed in the present study, which show the actual consumption of UPF, have policy implications. Higher taxes imposed on unhealthy foods in countries with higher consumption of UPF have been associated with a decline in their purchase as well as long-term health impacts by reducing obesity and the incidence of diabetes^(52–56)^. A few countries have also tried to control the health effects of UPF by setting limits on sugar, sodium, trans-fats, etc. front-of-package warning labels, regulating the marketing of UPF, especially for children, and controlling access to UPF in schools^(57–60)^. Thus, fiscal measures such as the combined effects of applying taxes on unhealthy foods along with subsidies on healthier foods, effective nutrition labelling and behaviour change communication strategies can help us to leapfrog the predictable trend of nutrition transition^(11,58–60)^.

The present study has a few limitations. First, for concurrent criterion validation of the screener, a 24-h dietary recall was used, which is prone to recall and reporting biases^(61)^. Second, this tool was only validated for the adult population; further validation on other age groups is warranted for the population-level data collection on UPF consumption. Although the sample size (*n* 304) was adequate to identify correlations between the Nova-UPF score and the UPF per cent share in the total caloric intake, the concurrent criterion validation study did not intend to stratify the sample further according to the socio-demographic and economic profile of the study population. Therefore, score performance according to socio-demographic characteristics may be further evaluated. Our study also has several strengths. It is the first study that aims to develop and validate a quick screener for UPF consumption tailored to the Indian context. The tool is easy, simple, has low participant burden, takes 6 min to administer and appropriately captures UPF consumption among a diverse population in different states of India. It can also be used for monitoring trends in UPF intake over time and informing policies.

### Conclusion

This study focused on the validation of the adapted Nova-UPF Screener (for India), which is a quick, simple and easy-to-administer dietary assessment tool for capturing UPF consumption among Indian adults. The Nova-UPF Screener (for India) collects and calculates the scores quickly and practically, which has a great potential to monitor the rising trend of UPF consumption among Indian adults^(11)^. To the best of our knowledge, the tool will be the first of its kind in India. This validated tool can be effectively implemented to add to the limited evidence on the actual consumption of UPF in diverse regions and among the different socio-economic strata in India. The objective estimates of UPF consumption among Indian adults using this screener can provide critical evidence on UPF intake trends over time, which may inform policies aimed at addressing the increasing burden of DR-NCD by developing and implementing interventions around creating healthier food environments. This tool can also be an effective instrument for documenting trends in UPF consumption objectively, thereby monitoring the efforts of the Government in addressing the food environment transition in India.

## Supporting information

Ghosh-Jerath et al. supplementary material Ghosh-Jerath et al. supplementary material

## Supplementary material

For supplementary material/s referred to in this article, please visit https://doi.org/10.1017/S0007114525105230
