# Supplementary material for: Validation of a screener to assess ultra-processed food consumption in the adult Indian population: the Nova-UPF Screener (for India)
Source: Br J Nutr. 2025 Sep 26;134(9):743–54. doi: 10.1017/S0007114525105230 (PMC12766108; doi:10.1017/S0007114525105230)
Supplement: Ghosh-Jerath et al. supplementary material [file S0007114525105230sup001.docx]

**Supplementary Sheet**

**Table 1: Questions asked from experts in regional consultations for content validation of the screener.**

| **S. No.** | **Questions** |
| --- | --- |
| 1. | Focusing just on the beverages and drinks (section A), do the existing categories seem appropriate to meet the objectives of this study? |
| 2. | Do you think any sub-categories of beverages need to be added, removed, or merged? |
| 3. | Are there any other regional traditional beverages that are ultra-processed that can be added to the list? |
| 4. | Focusing just on the foods in section B, do the existing categories seem appropriate to meet the objectives of this study? |
| 5. | Do you think any sub-categories of foods need to be added, removed, or merged to section B? |
| 6. | Are there any other regional traditional foods that are ultra-processed that can be added to section B? |
| 7. | Focusing just on the foods in section C, do the existing categories seem appropriate to meet the objectives of this study? |
| 8. | Do you think any sub-categories of foods need to be added, removed, or merged to section C? |
| 9. | Are there any other regional traditional foods that are ultra-processed that can be added to section C? |
| 10. | Overall, are the food items in each of the sub-categories sequenced appropriately? |
| 11. | Do you think including brand names is helpful? What can be the pros and cons of including brand names? |
| 12. | Do you think we should prepare a flipbook that will include the reference pictures for each UPF subcategory? |
| 13. | Do you think we should ask the respondents which food item they ate/drank when they tick a UPF subcategory so that we can correctly identify whether the food was UPF? |
| 14. | Is the language of the Tool appropriate? What other languages would you recommend the Tool be translated into? |
| 15. | What are your thoughts about the length of the Tool? |
| 16. | Any other feedback to share |

**Table 2: Questions asked from experts in the national consultation for content validation of the screener.**

| **S. No.** | **Questions** |
| --- | --- |
| 1. | Does the screener accurately reflect UPF consumed by the Indian population? |
| 2. | What is the feasibility of administering the screener as part of a national nutrition survey? |
| 3. | How should this tool be implemented, face-to-face, self-administered, online – in a national nutrition survey? |
| 4. | What should be the language of the screener (regional languages, Hindi, English)? |
| 5. | Any other feedback to share |

**Table 3: Face validation questions (objective assessment) captured using a questionnaire**

| **S. No.** | **Question** | **Answer** | |
| --- | --- | --- | --- |
| 1. | Were the instructions given by the interviewer clear? | [ ] Yes | [ ] No |
| 2. | Could the interviewer explain the sub-categories clearly? | [ ] Yes | [ ] No |
| 3. | Were the pictures shown for each sub-category clear? | [ ] Yes | [ ] No |
| 4. | Could you link the foods/drinks under each sub-category with the pictures? | [ ] Yes | [ ] No |
| 5. | Were the examples given in each sub-category enough? | [ ] Yes | [ ] No |
| 6. | Did the examples under each sub-category include foods and drinks from your region of India? | [ ] Yes | [ ] No |
| 7. | Do you think taking brand names helped in identifying/recalling the foods/drinks easier? | [ ] Yes | [ ] No |
| 8. | Was the length of the screener appropriate? | [ ] Yes | [ ] No |

**Table 4: Input from regional consultation experts on the draft Nova-UPF Screener (for India); Section A – Beverages; Section B - Packaged meals and savoury snacks; Section C - Desserts and accompaniments.**

**Section A – Beverages**

| **Section A – Beverages** | | | | |
| --- | --- | --- | --- | --- |
| **S.No.** | **Sub-category** | **Inputs from regional experts** | **Inputs accepted** | **Inputs not accepted** |
| **A1.** | Aerated cold drinks/soft drinks; Diet drinks | Aerated cold drinks/soft drinks; Diet drinks; Flavoured water (like black water, volcanic water, sparkling water, alkaline water); alcohols and alcoholic beverages; Goli Soda | - Flavoured water (like fruit flavoured water) | **Non UPFs**   - Black water, volcanic water, sparkling water, alkaline water   **UPFs**   - Goli Soda - Alcohols and alcoholic beverages |
| **A2.** | Energy drinks | Energy drinks; Sports drinks | Sports drinks | - |
| **A3.** | Packaged, and branded fruit-based bottled drinks and concentrates (like tetra pack fruit juices, concentrates, powdered juices); Packaged, and branded vegetable juices; Iced- tea | Packaged, and branded fruit-based bottled drinks and concentrates (like tetra pack fruit juices, concentrates, powdered juices); Packaged, and branded vegetable juices; Iced-tea; Canned/Bottled/Powdered Tea and Coffee; Kahwa mix; Coconut water; Sherbets, thandai, shikanji, chandan, kevada, khas, jal jeera, kairi/aam panna, fruit premixes, sugarcane juice, aloe vera juice, jackfruit drinks, gooseberry drinks, cashew apple drinks, rose syrup | - Canned/Bottled/ Powdered Tea and Coffee - Sherbets, thandai | **Non UPFs**   - Kahwa mix - Sugarcane juice - Cashew apple drinks   **UPFs**   - Shikanji, chandan, kevada, khas, jal jeera, kairi/aam panna, rose syrup - Coconut water - Aloe vera juice - Gooseberry drinks |
| **A4.** | Packaged, and branded flavoured milk, milk products and milk substitutes (like chocolate milk, cold | Packaged, and branded flavoured milk, milk products and milk substitutes (like chocolate milk, cold coffee, flavoured lassi, buttermilk, probiotic drinks, casein hydrolysates, | - Protein powders - Milk whiteners - Flavoured coffee powders | **Non UPFs**   - Casein hydrolysates - Chukku coffee |
|  | coffee, flavoured lassi, buttermilk, probiotic drinks, milk-based powdered health drinks, soymilk, rice milk, almond milk) | protein powders, smoothies, milk- based powdered health drinks, soymilk, rice milk, almond milk, coconut milk, camel milk, goat milk, walnut milk, oat milk, peanut milk); Milk whiteners; Coconut milk powder; flavoured coffee powders (such as hazelnut, vanilla, etc.), chukku coffee, masala chai, iced tea, ragi malt, sattu malt mixes; Liquid fruit custard | - Masala chai - Iced tea | - Sattu malt mixes - Liquid fruit custard   **UPFs**   - Smoothies - Coconut milk, camel milk, goat milk, walnut milk, oat milk, peanut milk   Coconut milk powder |
| **A5.** | Packaged and flavoured Yogurt; Mishti doi; Kheer | Packaged and flavoured Yogurt; Greek yogurt; Fruit yogurt with chunky granola; Mishti doi; Kheer; Payasam; Rabdi; Basundi; Shrikhand | - Fruit yogurt - Payasam | **Non UPFs**   - Shrikhand - Rabdi   **UPFs**   - Greek yogurt - Fruit yogurt with chunky granola - Basundi |

Note - New foods suggested during content validation are in red.

**Section B – Packaged meals and savoury snacks**

| **Section B – Packaged meals and savoury snacks** | | | | |
| --- | --- | --- | --- | --- |
| **S.No.** | **Sub-category** | **Inputs from regional experts** | **Inputs accepted** | **Inputs not accepted** |
| **B1.** | Packaged, and branded bread (like pao, hot dog buns, burger buns, pizza base, tortillas); Packaged branded Bread mix; Puff pastry sheets; Spring roll sheets | Packaged, and branded bread (like pao, hot dog buns, burger buns, pizza base, tortillas); Packaged branded Bread mix; Puff pastry sheets; Spring roll sheets; Rice paper sheets; Lasagna sheets | - | **Non UPFs**   - Rice paper sheets - Lasagna sheets |
| **B2.** | Packaged, and branded rotis, naans, parathas, kulchas | Packaged, and branded rotis, naans, parathas (plain or stuffed), kulchas, khakra, puran poli, bajra rotla, missi roti | - Khakra | **Non UPFs**   - Puran poli - Bajra rotla - Missi roti |
| **B3.** | Packaged and branded instant soup; Instant noodles; Instant/ ready-to-eat pasta/ poha/ upma | Packaged and branded instant soup; Instant noodles; Rice noodles; Vermicelli; Instant/ ready-to-eat pasta/ poha/ upma; Instant savoury oats, Instant dalia, Instant sambar, Instant rasam, Instant idiyappam, Puttu flour; Ready-to-eat haleem | - Instant savoury oats | **Non UPFs**   - Instant sambar - Instant rasam - Instant idiyappam - Puttu flour - Ready-to-eat haleem   **UPFs**   - Rice noodles - Vermicelli - Instant dalia |
| **B4.** | Packaged, and branded idli, dosa, vada mix | Packaged, and branded idli, dosa, ragi dosa, vada, dhokla, handwa, dahi vada, uttapam, bedmi puri, moong dal pakodi, pongal, adai, pesarattu, besan cheela, upma mix, ready-to-eat/cook curry mixes like paneer butter masala, vegetable curry, rice paste/ powder mixes such as puliyogare, vangi bath, bisibele bath mix | - Dhokla - Dahi vada - Rice paste/ powder mixes such as puliyogare, vangi bath, bisibele bath mix | **Non UPFs**   - Bedmi puri - Pongal - Adai - Pesarattu - Besan cheela   **UPFs**   - Handwa - Uttapam - Moong dal pakodi - Upma |
| **B5.** | Packaged, and branded breakfast cereals; Cereal bars (like granola bars, energy bars) | Packaged, and branded extruded breakfast cereals (like cornflakes, muesli, chocos, granola, rice crispies, ragi bites, ragi flakes); Cereal bars (like granola bars, energy bars) | - Chocos - Ragi bites | **Non UPFs**   - Cornflakes - Ragi flakes   **UPFs** |
|  |  |  |  | - Muesli - Granola - Rice crispies |
| **B6.** | Packaged, and branded cookies, biscuits, cream biscuits; Cream puffs/rolls; Cream croissants; Rusks | Packaged, and branded cookies, biscuits, cream biscuits; Cream puffs/rolls; Cream croissants; Rusks; Nankhatai; Khari puffs; Karachi biscuits; Cake slices | - | **UPFs**   - Nankhatai - Khari puffs - Karachi biscuits - Cake slice |
| **B7.** | Packaged, and branded chips (plain and flavoured); Nachos; Puffs and cheeseballs; Pop-corn | Packaged, and branded chips (plain and flavoured); Nachos; Puffs and cheeseballs; Pop-corn; Extruded fryums, Banana chips | - | **Non UPFs**   - Banana chips   **UPFs**   - Extruded fryums |
| **B8.** | Packaged, and branded Indian namkeens (like bhujia, mixtures, papads, flavoured makhana) | Packaged, and branded Indian namkeens (like bhujia, mixtures, papads, flavoured makhana, boondi, chakli, murukku); Flavoured khakhra, tanatan, gathiya, fafda, matharis, chiwada, small kachoris, samosas, nipattu, khara sev, flavoured nuts, roasted chana and namkeens  (cereal-pulse mixes), soya sticks | - Murukku - Flavoured nuts | **Non UPFs**   - Boondi - Gathiya - Fafda - Matharis - Small kachori - Samosas - Nipattu - Khara sev - Roasted chana and namkeens (cereal-pulse mixes) - Soya sticks   **UPFs**   - Chakli - Flavoured khakhra - Tanatan - Chiwada |
| **B9.** | Packaged, branded, and frozen non- vegetarian foods (like chicken nuggets, burger patty, samosa, cutlets, non-veg pizza); Packaged, branded and frozen meats preparations (like marinated tandoori chicken, prawns snacks, fish snacks, sausages, salami) | Packaged, branded, and frozen non- vegetarian foods (like chicken tikka, chicken nuggets, kebabs, burger patty, samosa, cutlets, non-veg pizza, non-veg momos); Packaged, branded and frozen meats preparations (like marinated tandoori chicken, meat balls, prawns snacks, fish snacks, sausages, salami); Smoke dried fish, Masor Tenga, Dry fish powder | - Chicken tikka - Kebabs - Non-veg momos | **Non UPFs**   - Masor tenga - Dry fish powder   **UPFs**   - Meat balls - Smoke dried fish |
| **B10.** | Packaged, branded and frozen vegetarian snacks (like french fries, vegetarian burger patty, aloo tikki, samosas, veg pizza, spring rolls) | Packaged, branded and frozen vegetarian snacks (like french fries, vegetarian burger patty, aloo tikki, potato shots, potato wedges, mashed potato powder, samosas, veg pizza, veg momos, spring rolls, appam, bonda, idli, cheese puffs, cheesecorn nuggets) and paneer based snacks | - Paneer based snacks | **Non UPFs**   - Mashed potato powder - Appam - Idli   **UPFs**   - Potato shots - Potato wedges - Veg momos - Bonda - Cheese puffs - Cheesecorn nuggets |
| **B11.** | Packaged, branded and frozen or ready-to-eat meals (like palak paneer, rajmah-rice, puloa, biryani, dal makhani, chicken curry) | Packaged, branded and frozen or ready-to-eat meals (like palak paneer, rajmah-rice, puloa, biryani, dal makhani, chicken curry, chokhi- dhani meal, gatta curry vegetables, curry pakodi meal, sambar-rice, rasam, tamarind rice, bisibele bath) | - Bisibele bath | **Non UPFs**   - Chokhi-dhani meal - Gatta curry vegetables - Tamarind rice   **UPFs**   - Curry pakodi meal - Sambar-rice - Rasam |
| **B12.** | Packaged, and branded ketchup, chutneys, sauces, purees; | Packaged, and branded ketchup, chutneys, sauces, pasta-pizza sauce, sriracha sauce, purees; Packaged, and branded pickles; Kharissa | - Pasta-pizza sauce - Veg and Non-veg pickles (Kharissa, beef, fish and | **Non UPFs**   - Golgappa paani powder |
|  | Packaged, and branded pickles | (bamboo shoot pickle); Beef, fish and pork pickles, Gongura pickle; Drumstick pickle; Instant seasonings/taste-makers; Instant gravies/curry paste; Ginger-garlic paste; Garlic chutney; Bhelpuri chutney; Golgappa paani powder | pork pickles, gongura pickle, drumstick pickle)   - Instant seasonings/taste- makers - Instant gravies/curry paste - Ginger-garlic paste | **UPFs**   - Sriracha sauce - Garlic chutney, bhelpuri chutney |

Note - New foods suggested during content validation are in red.

| **Section C - Desserts and accompaniments** | | | | |
| --- | --- | --- | --- | --- |
| **S.No.** | **Sub-category** | **Inputs from regional experts** | **Inputs accepted** | **Inputs not accepted** |
| **C1.** | Packaged, and branded cakes, muffins, or desserts; Packaged, and branded dessert mixes (like cake, pancake, brownie, jelly, custard, gulab jamun, barfi) | Packaged, and branded cakes, muffins, or desserts (like gajar halwa, soanpapadi, laddus, chikkis, rasogulla, sandesh); Packaged, and branded dessert mixes (like cake, pancake, brownie, jelly, custard, gulab jamun, barfi, rabdi, donuts, waffles, bagels, mohanthal, kesari, payasam, porridge, sattu, jalebi, icecream, china grass) | - Soanpapadi | **Non UPFs**   - Chikkis, rasogulla - Mohanthal, kesari, porridge, sattu, china grass   **UPFs**   - Gajar halwa, laddus - Rabdi, donuts, waffles, bagels, payasam, jalebi, icecream |
| **C2.** | Packaged, and branded fruit-based preserves (like jams, jellies, murabba) | Packaged, and branded fruit-based preserves (like jams, jellies, murabba), Marmalade, amla candy, pineapple candy, dehydrated colored fruit candies, fruit leather (aam papad), fruit candies and flavoured candy bars, palm jaggery, palm sugar/mishri/candies. | - Marmalade - Fruit leather (aam papad) fruit candies | **Non UPFs**   - Amla candy - Palm jaggery - Palm sugar/mishri/candies   **UPFs**   - Pineapple candy - Dehydrated colored fruit candies - Flavoured candy bars |
| **C3.** | Packaged, and branded ice-creams and flavoured ice-bars | Packaged, and branded ice-creams and flavoured ice-bars, Yoghurt and mishti doi, Gelato, Kulfi, Frozen desserts | - Kulfi | **Non UPFs**   - Gelato - Frozen desserts   **UPFs**   - Yoghurt - Mishti doi |
| **C4.** | Chocolates; Toffees; Lollipops; Chewing gums | Chocolates; Toffees; Lollipops; Chewing gums; Flavoured mouth fresheners, paan churries, paan masala; Marshmallows; Chewies; Sprinklers | - Flavoured mouth fresheners | **Non UPFs**   - Chewies   **UPFs**   - Paan churries - Marshmallows - Sprinklers   Paan masala |
| **C5.** | Chocolate spreads; Chocolate syrups | Chocolate spreads, Hazelnut spread; Toppings like gulkand, chocolate syrups, flavoured syrups such as raspberry syrups, strawberry syrups, maple syrup, date syrup, caramel syrup, honey, thandai | - Hazelnut spread | **Non UPFs**   - Gulkand - Date syrup - Honey   **UPFs**  Flavoured syrups such as raspberry syrups, strawberry syrups, maple syrup, caramel syrup, thandai |
| **C6.** | Packaged, and branded milk-based spreads, dip and cheese (like cheese slices/cubes, cheese spread, mayonnaise, cheese dips, sandwich spreads); Packaged, and branded peanut spread/ butter | Packaged, and branded milk-based spreads, dips and cheese (like cheese slices/cubes, cheese spread, mayonnaise, cheese dips, sandwich spreads); Packaged, and branded peanut spread/butter, coconut butter, almond butter; Condensed milk; Salad dressings; Fresh cream | - Salad dressings | **Non UPFs**   - Coconut butter - Almond butter - Condensed milk   Fresh cream |
| **C7.** | Margarine; Packaged, and branded flavoured butter (like garlic butter) | Margarine; Lard; Packaged, and branded flavoured butter (like garlic butter, bacon butter); Coconut cream | - Coconut cream | **Non-UPFs**   - Lard   Bacon butter |

Note - New foods suggested during the content validation are in red
